# Multi-Tissue Transcriptomic Profiles of Tarim and Tianshan Red Deer Under Common Conditions

**DOI:** 10.3390/vetsci13101066

**Published:** 2026-10-09

**Authors:** Xilai Zhao, Nursat Turxunjan, Xu Zhou, Xinlong Hao, Xiaowei Jia, Linzhi Yan, Miaohua Wang, Zhen Li, Wenxi Qian

**Affiliations:** 1 College of Life Science and Technology, Tarim University, Alar 843300, China; 19954264910@163.com; 2 College of Animal Science and Technology, Tarim University, Alar 843300, China; 15559464033@163.com (N.T.);; 3 Tarim Animal Husbandry Science and Technology Key Laboratory of Xinjiang Production and Construction Corps, Alar 843300, China

**Keywords:** red deer, multi-tissue transcriptome, ruminant digestive physiology, rumen, RNA sequencing, gene expression

## Abstract

We profiled 132 RNA-sequencing libraries representing 11 tissues from six Tarim and six Tianshan adult male red deer maintained under common feeding and management. Tissue identity dominated expression structure among 16,308 expressed genes. Across three differential-expression model specifications, 1253 robust group-associated gene–tissue pairs were identified, involving 1184 genes and concentrated in rumen and rectum. Directional enrichment returned 77 terms meeting both within-set and study-wide false-discovery thresholds. Tianshan-group rumen genes were enriched for cytoskeletal, adhesion, ion-transport and circadian annotations, whereas Tarim-group reticulum genes were enriched for cell-surface and immune-signaling annotations. Cross-tissue recurrence was uncommon and identified a small set of annotated genes and provisional loci for follow-up. Complementary whole-genome principal component analysis (PCA) separated the recorded groups across two genotype-depth filters; weighted Weir–Cockerham *F*_ST_ was 0.341 and 0.325. These results describe tissue-resolved expression patterns and genetic structure in the sampled cohort. Functional, taxonomic and population-wide interpretations require independent validation.

## Simple Summary

We compared gene expression across 11 tissues from 12 adult male red deer maintained on the same diet and under the same management. Tissue identity explained most transcriptomic variation, while expression differences between the recorded Tarim and Tianshan source groups were concentrated in selected digestive tissues, especially the rumen. Whole-genome data from the same animals provided complementary evidence of genetic structure. The findings identify candidates for follow-up studies of forestomach biology and do not by themselves demonstrate improved health or local adaptation.

## 1. Introduction

We use Tarim group (TRD) and Tianshan group (TSD) as operational labels for the two recorded source stocks of *Cervus* deer. Earlier studies used *Cervus hanglu yarkandensis* or *Cervus elaphus yarkandensis* for Tarim deer and *Cervus canadensis songaricus* or *Cervus elaphus songaricus* for Tianshan deer [1,2,3,4]. Taxonomic treatments differ among authorities; these names are cited as literature usage and are not formal assignments of the 12 study animals. No external taxonomic reference panel was applied, and hybrid ancestry was not tested. The source-stock comparison is therefore described using group labels throughout.

Published field studies describe distinct ecological settings and seasonal foraging contexts for Tarim and Tianshan deer. In Tarim Basin field samples, diet metabarcoding reported seasonally varying use of poplar and other plants, with cotton also detected [5]. A winter diet study from the Nanshan Mountains near Urumqi reported frequent use of spruce, grasses, sedges, and bryophytes by Tianshan deer [6]. These studies describe regional free-ranging animals and cannot be assumed to represent the origin, lifetime diet, or habitat of the farmed animals studied here. All study animals received the same ration during the 90-day common-management period. This context motivates a comparison of organ-level expression, while leaving genetic, developmental, and prior environmental contributions unresolved. These botanical observations motivate nutritional questions about forage fiber, digestible energy, and protein supply, but do not quantify nutrient intake in the farmed study animals. The current experiment therefore reports the shared experimental ration in Table 1 rather than assigning regional dietary nutrient values to either source group.

Multi-tissue transcriptomic studies in humans and cattle have mainly mapped organ expression and regulatory variation [7,8], while sheep and dairy-cattle studies have described gastrointestinal specialization or changes associated with lactation [9,10]. These studies provide a framework for comparing tissue identity, but they do not compare Tarim and Tianshan source stocks under common rearing conditions. Forestomach compartments differ in mechanical function, epithelial physiology, fermentation exposure, and immune surveillance, while the intestines contribute region-specific absorptive and mucosal functions [9,10,11,12,13,14]. The specific gap addressed here is whether group-associated expression differs among these organs in the same animals when recent feeding and management are shared.

We asked how expression variance was distributed among tissue, animal, source group, and group-by-tissue components; which within-tissue contrasts were robust across model specifications; and whether differentially expressed genes or annotation-based functional units recurred across tissues. We expected tissue identity to dominate globally and group-associated differences to be concentrated in selected digestive compartments. Whole-genome sequencing (WGS) data from the same animals provided an independent description of genetic structure, without an external taxonomic panel or an ancestry classifier. Inferences are restricted to the sampled cohort.

## 2. Materials and Methods

### 2.1. Animals, Housing, and Diet

Twelve clinically healthy males aged 2.5–3.0 years were studied, comprising six animals in each recorded source group. The Tarim group was bred and maintained at the Tarim University Red Deer Breeding Base in Alar, Xinjiang, China. The Tianshan group came from a breeding farm operated by Xinjiang Oasis Deer Island Agricultural Technology Co., Ltd., at No. 138, Dabancheng Village, Dabancheng Town, Dabancheng District, Urumqi, Xinjiang, and arrived at the university base on 25 July 2025. The Tianshan animals first underwent a seven-day environmental-acclimation period. All 12 animals were then individually housed, pre-fed together for 10 days, and maintained on the same experimental ration and management for 90 days. Animals with external injury, gastrointestinal disease, or evidence of suboptimal health were excluded. Individual birthplaces, complete earlier husbandry histories, and a complete pedigree were not available. Possible within-farm relatedness was not excluded. RNA and DNA libraries were paired according to the animal-level records confirmed by the authors. The study metadata and model design are provided in Table S1.

A total mixed ration was offered ad libitum twice daily at 10:00 and 18:00, with unrestricted access to water. Residual feed was removed before the next feeding. The concentrate-to-roughage ratio was 3:7 on an air-dry basis. Ingredient composition and calculated nutrient levels are presented in Table 1.

### 2.2. Slaughter and Tissue Collection

All deer were slaughtered on 10 November 2025 between 08:00 and 12:00. The order alternated between recorded source groups, beginning with a Tarim animal, to reduce confounding of group with collection time. Feed was withdrawn for 6 h; water remained available. Xylazine hydrochloride (2 mg kg^−1^; Dunhua Shengda Animal Pharmaceutical Co., Ltd., Dunhua, China) was administered intravenously using the same protocol. After deep unconsciousness was confirmed by loss of nociceptive responses and the corneal reflex, carotid exsanguination was performed.

Eleven tissues were collected from every animal: rumen (RUM), reticulum (RET), omasum (OMA), abomasum (ABO), duodenum (DUO), jejunum (JEJ), ileum (ILE), cecum (CEC), colon (COL), rectum (REC), and liver (LIV). Gastrointestinal samples comprised full-thickness wall. All rumen samples were taken from the middle of the ventral sac, avoiding the dorsal sac, cranial and caudal blind sacs, and the margins of ruminal pillars. The rumen was opened along the outer greater curvature, its contents were removed, and the inner wall was briefly rinsed with precooled RNase-free phosphate-buffered saline. Duodenal samples were obtained from the mid-region distal to the pylorus, jejunal samples from the middle portion of the jejunum, and ileal samples proximal to the ileocecal valve. Exact measured distances and finer landmarks for the other gastrointestinal sites were not recorded. Liver parenchyma was collected internally while avoiding the capsule, large vessels, bile ducts, and visibly damaged regions.

Following anesthesia, exsanguination, and abdominal inspection, the liver was sampled first. The stomach and gastrointestinal tract were then removed and sampled in the following sequence: rumen, reticulum, omasum, abomasum, duodenum, jejunum, ileum, cecum, colon, and rectum. RNA-sequencing tissue specimens were immediately snap-frozen in liquid nitrogen after excision, subsequently stored at −80 °C, and shipped on dry ice to Biomarker Technologies (Beijing, China). The elapsed time from excision to freezing was not recorded quantitatively. Fixed tissue preparations from other experimental work were not used for RNA sequencing.

### 2.3. RNA Extraction, Library Preparation, and Sequencing

The organ-level analysis used 132 paired-end RNA-sequencing libraries, representing 11 tissues from each of the 12 animals. The complete sequencing project contained 156 libraries; the other 24 represented the rumen muscular layer and rumen papilla from the same animals and were outside this organ-level analysis.

Biomarker Technologies extracted total RNA using TRIzol™ Reagent (Invitrogen, Thermo Fisher Scientific, Waltham, MA, USA; 100 mL; Cat. No. 15596026). RNA concentration and purity were assessed by NanoDrop, and integrity by an Agilent 2100 Bioanalyzer or LabChip GX system. The final quality records for the 132 analyzed samples contained RNA integrity number (RIN) values ranging from 3.5 to 9.2 (median 7.9); four had an RIN below 5. Across the full 156-sample project, the authors confirmed that 146 extractions succeeded in the first round, eight in the second, and two in the third, with all final preparations used for library construction. Re-extraction rounds did not constitute additional biological samples. A single numerical provider acceptance cutoff was not documented; therefore, the measured final RIN values are a reported rather than an inferred threshold. Final sample-level quality records, including the distinction between the 132 analyzed and 24 additional libraries, are provided in Table S11.

One microgram of total RNA per sample was used to construct libraries with the NEBNext Ultra RNA Library Prep Kit for Illumina (New England Biolabs, Ipswich, MA, USA; the specific catalog number was not retained in the available laboratory records). Polyadenylated RNA was enriched with oligo(dT) beads, fragmented, and reverse-transcribed. Following second-strand synthesis, end repair, adaptor ligation, size selection of approximately 240 bp fragments, and PCR enrichment, libraries were assessed on an Agilent 2100 Bioanalyzer. Paired-end 150 bp sequencing was conducted on an Illumina NovaSeq X Plus platform. Provider-filtered paired FASTQ files were used for downstream analysis; no second read-filtering step was applied locally. Each library yielded at least 5.75 Gb of clean data, with Q30 values of at least 95.59%.

### 2.4. Transcript Quantification and Gene Filtering

Reads were quantified against the CEY_v1 Tarim red deer assembly (NCBI accession GCA_010411085.1) and Ensembl release 116 annotation. A transcriptome index containing 46,855 annotated transcripts and a partial genomic decoy was generated. Salmon v1.10.3 [15] was run with automatic library-type detection and selective-alignment validation (validateMappings). Transcript-level estimates were summarized to genes with tximport [16]. Genes with transcripts per million (TPM) ≥ 1 in at least six libraries were retained, resulting in 16,308 expressed genes. Count-scale data were normalized by the trimmed mean of M-values method [17] implemented in edgeR [18]; log_2_(TPM + 1) values were used for exploratory expression analyses.

### 2.5. Global Expression Structure and Variance Decomposition

Principal component analysis (PCA) was performed on the 5000 genes with the greatest variance across log_2_(TPM + 1) values. Pairwise sample similarity was summarized by Spearman correlation. For each expressed gene, variance was partitioned among tissue, population, animal, population-by-tissue interaction, sequencing-run class, and residual components using mixed-effects modeling. Variance fractions were summarized across genes by the median, interquartile range, and 5th–95th percentile. The proportion of genes for which each component explained >5% of total variance was also calculated. Existing within-tissue PCA scores, calculated separately for each tissue using 2000 genes and all 12 animals, were also plotted. Colors indicated the recorded source group, point shapes indicated sequencing run, and animal labels A–L identified the paired individuals (Figure S1). The run labels were joined to the same study metadata used in the differential-expression models: 129 libraries belonged to run 592, two to run 595 and one to run 610. The model run-class variable distinguished the predominant run from supplemental runs; the Supplementary Figure distinguishes the three individual run numbers. The PCA coordinate tables and run metadata are supplied in Supplementary Dataset S3. Variance-component summaries are provided in Table S10.

### 2.6. Tissue-Resolved Differential Expression

Population contrasts were estimated within each tissue using voom precision weights [19] and the dream mixed-model framework [20], with empirical-Bayes moderation from limma [21] and implementation in variancePartition [22]. The primary model was ~0 + population:tissue + run class + (1|animal). Two sensitivity models were fitted: the same population-by-tissue model without run class across all 132 samples, and the no-run-class model restricted to the predominant sequencing-run class (129 samples). Contrasts were oriented as Tianshan minus Tarim; positive log_2_ fold changes therefore denote higher expression in Tianshan red deer.

A gene–tissue pair was classified as a robust differentially expressed gene (DEG) when |log_2_ fold change| was ≥1 and the Benjamini–Hochberg false-discovery rate (FDR) [23] was ≤0.05 in all three models, with a concordant direction of effect. One gene could not be fitted successfully in any of the 11 tissue-specific contrasts (11 failed gene–tissue fits in total); these fits were excluded from DEG classification and were not counted as evidence of no difference. Robust pairs are listed in Table S2; complete primary-model estimates are supplied in Supplementary Dataset S1.

### 2.7. Directional Functional Enrichment

Robust DEGs were separated by tissue and direction before Gene Ontology (GO) enrichment [24]. Gene identifiers were connected through the Ensembl annotation and UniProt knowledgebase [25], and pathway terms were obtained from the Kyoto Encyclopedia of Genes and Genomes (KEGG) [26]. Terms were tested against the expressed-gene background. Significance required both a within-set FDR (SetFDR) ≤ 0.05 and an FDR corrected across all tissue, direction, and database tests (StudywideFDR) ≤ 0.05. Closely related records mapping to the same UniProt entry were collapsed into functional units to prevent identifier redundancy from inflating recurrence. Significant terms are listed in Table S3; complete annotations and enrichment tests are supplied in Supplementary Dataset S2.

### 2.8. Cross-Tissue Recurrence Analysis

For each functional unit, we counted the number of tissues in which robust differential expression occurred in a consistent direction. Tissue-pair similarity was calculated as the Jaccard index, the intersection divided by the union of robust functional-unit sets. Units with discordant directions across tissues were cataloged separately and excluded from the consistent-direction heatmap. Recurrence summaries and the direction-conflict audit are provided in Tables S6–S9.

Genomic coordinates of the unnamed recurrent CEY_v1 loci were extracted from the Ensembl release 116 GTF annotation and reported as 1-based inclusive intervals. Eight corresponding public reference proteins were queried against the NCBI nonredundant protein database (nr) using BLASTP 2.17.0+ on 4 October 2026, with an E-value cutoff of 1 × 10^−5^, BLOSUM62, low-complexity masking and composition-based adjustment. The first returned hit and the first hit with a named, non-hypothetical description were recorded separately, with accession, species, E-value, sequence identity and single-HSP query coverage (Table S12). Only publicly distributed Ensembl reference proteins were submitted. *SCARNA2* is noncoding and was not subjected to BLASTp. Similarity-based assignments were treated as provisional annotations; original gene identifiers were retained.

### 2.9. Statistical Analysis

Analyses were performed in R v4.5.2 using edgeR, limma, variancePartition, lme4, lmerTest, and BiocParallel. Differential-expression estimates used voom precision weights, dream mixed models, empirical-Bayes moderation, and Benjamini–Hochberg FDR control. A gene–tissue pair was retained at |log_2_ fold change| ≥ 1 and FDR ≤ 0.05 in all three model specifications with concordant direction. Enrichment was considered significant only when SetFDR and StudywideFDR were both ≤0.05. Figures were assembled from machine-readable source tables. WGS PCA used physical single-nucleotide polymorphism (SNP) thinning and mean imputation. The weighted Weir–Cockerham estimate was the ratio of summed per-site numerators to summed denominators, rather than the arithmetic mean of per-site *F*_ST_. KING estimates were descriptive; no family relationships were inferred from threshold categories.

### 2.10. Whole-Genome Resequencing Analysis

Joint variants were called from the 12 DNA resequencing BAM files against the CEY_v1 reference genome using bcftools v1.22 mpileup and multiallelic variant calling. Chromosomes 1–33 were processed in segments of at most 10 Mb with a 1 kb boundary buffer; segment outputs were trimmed to the nonoverlapping target intervals before concatenation. Autosomal genotypes for the same 12 animals were analyzed using the animal-level map linking each animal’s RNA and DNA libraries. Variants were restricted to chromosomes 1–33. Separate datasets with read-depth (DP) thresholds of ≥3 and ≥5 were constructed by treating genotypes below each read-depth threshold as missing. Candidate SNPs were required to be biallelic, have minor-allele frequency ≥0.05 and overall missingness ≤0.20, and have genotypes in at least five of six animals in each recorded group. For PCA, SNPs were physically thinned by retaining one SNP per 50 kb interval; missing genotypes were mean-imputed in PLINK2 v2.0.0-a.7.11. PCA was repeated after excluding T23001 because its genotype missingness was elevated in the DP ≥ 5 dataset. Weir–Cockerham *F*_ST_ was estimated between the two recorded groups across each full candidate SNP set, and the weighted estimate was calculated as the sum of per-site *F*_ST_ numerators divided by the sum of per-site denominators. KING pairwise estimates were used descriptively and did not alter the recorded sample assignments. No external ancestry reference panel was used. Summary statistics distinguishing full candidate sets from physically thinned PCA sets are provided in Table S13.

## 3. Results

### 3.1. Tissue Identity Dominates the Global Expression Landscape

**Figure 1:**
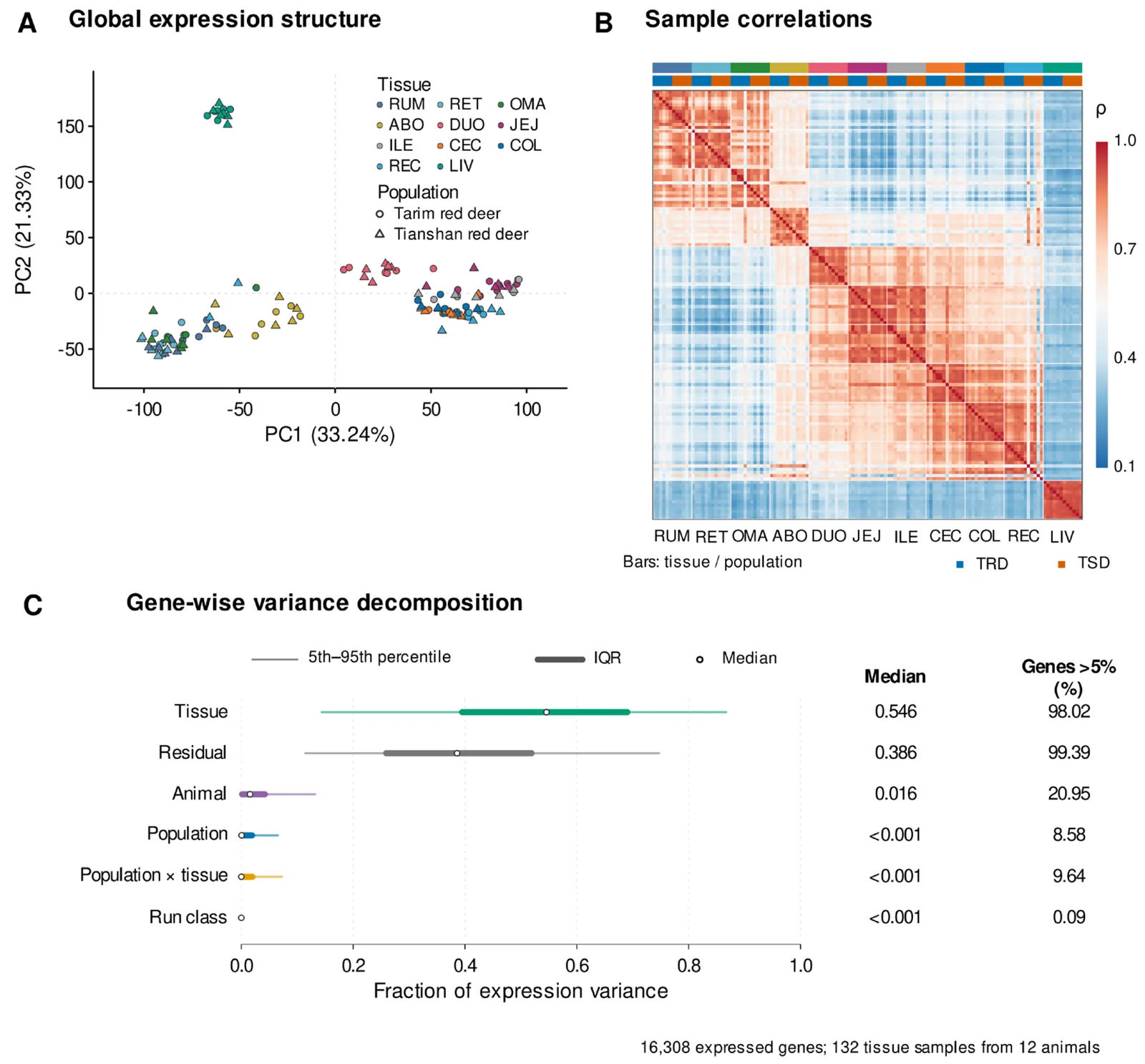
Global expression structure and gene-wise variance decomposition. (**A**) Principal component analysis of 132 tissue samples based on the 5000 most variable genes. Colors denote tissues and symbols denote source groups. (**B**) Spearman correlation matrix of log_2_(TPM + 1) expression values; annotation bars indicate tissue and source group. TRD and TSD denote the Tarim and Tianshan source groups, respectively. (**C**) Distribution of variance fractions across 16,308 expressed genes. Thin lines show the 5th–95th percentile, thick lines the interquartile range, and open circles the median. The table reports component medians and the percentage of genes for which a component explained >5% of total variance.

The final dataset comprised 132 libraries—11 tissues from each of the 12 animals—with no missing tissue–animal combination. After expression filtering, 16,308 genes were analyzed. Samples separated predominantly by anatomical origin: principal component 1 explained 33.24% of the expression variance and principal component 2 explained 21.33% (Figure 1A). The correlation matrix reinforced this structure, with coherent blocks corresponding to forestomach, intestinal, and liver samples; population labels were nested within these broader tissue-defined clusters (Figure 1B). Within-tissue RNA PCA is shown separately in Figure S1 with all sequencing runs marked; it supplements the global view without replacing the tissue-resolved statistical contrasts.

Gene-wise variance decomposition quantified the same hierarchy. Tissue accounted for a median fraction of 0.546 of expression variance and exceeded 5% for 98.02% of expressed genes. Residual variation had a median fraction of 0.386 and exceeded 5% for 99.39% of genes. The animal component was smaller (median, 0.016; >5% in 20.95% of genes). Median fractions for population and population-by-tissue interaction were both <0.001, although these components exceeded 5% for 8.58% and 9.64% of genes, respectively. Sequencing-run class contributed a median fraction <0.001 and exceeded 5% for 0.09% of genes (Figure 1C). Thus, group-associated regulation affected a minority of genes but was not uniformly distributed across tissues.

### 3.2. Group-Associated Expression Differences Are Concentrated in the Rumen and Rectum

**Figure 2:**
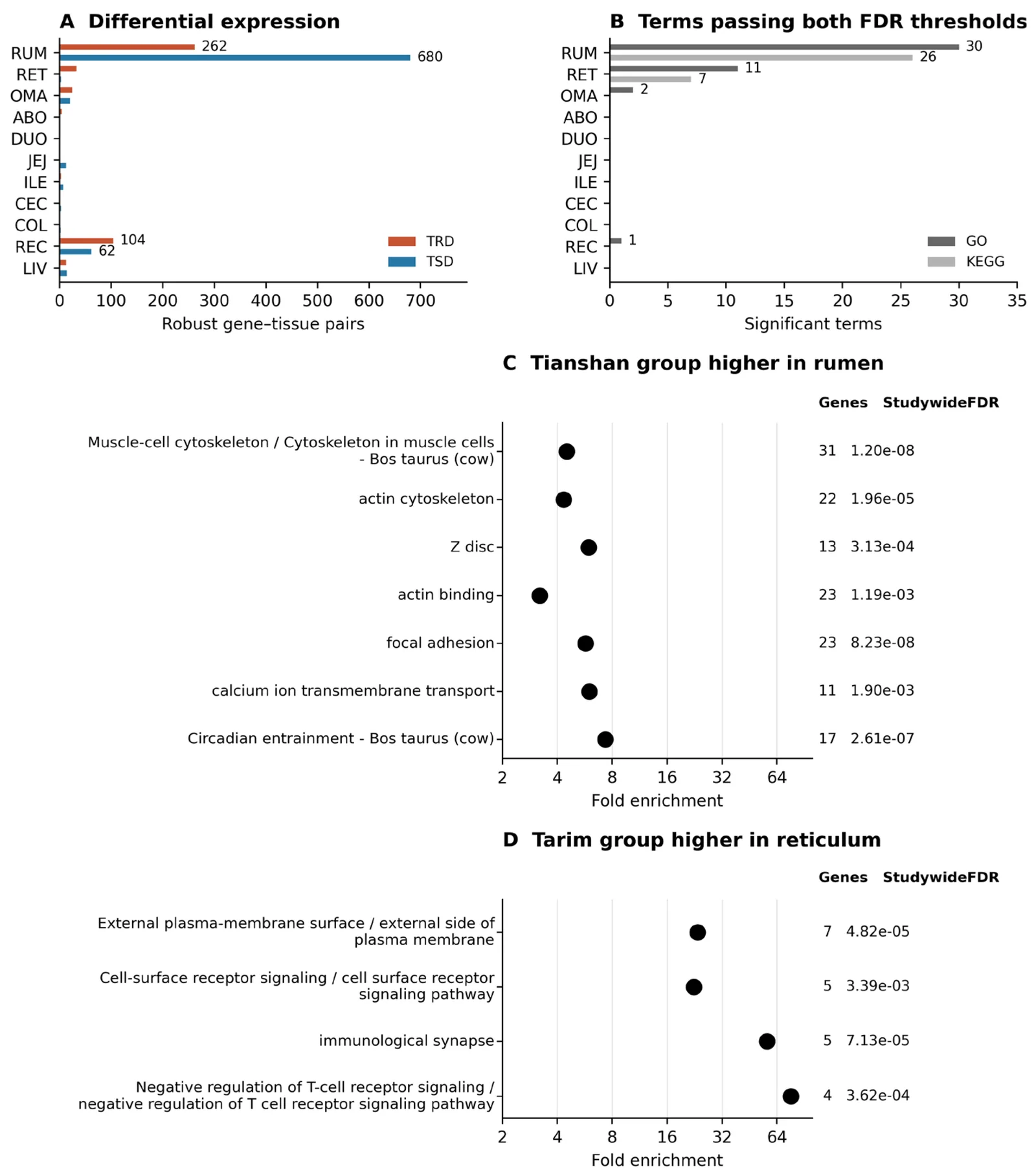
Tissue-resolved differential expression and functional enrichment. (**A**) Raw numbers of robust gene–tissue pairs on a linear scale. (**B**) GO and KEGG terms passing both SetFDR ≤ 0.05 and StudywideFDR ≤ 0.05. (**C**) Representative terms enriched among TSD-higher rumen genes. (**D**) Representative terms enriched among TRD-higher reticulum genes. Full source term names are retained alongside shortened labels where they differ; the right-hand columns give the overlapping gene count and StudywideFDR to three significant figures. For these 11 terms, overlapping gene counts equal the corresponding collapsed functional-unit counts. Fold enrichment is plotted on a base-2 logarithmic axis with raw fold-enrichment labels. TRD and TSD denote the Tarim and Tianshan source groups, respectively.

Across the three-model criterion, 1253 robust gene–tissue pairs involving 1184 genes were detected between the Tarim and Tianshan cohorts. Of these pairs, 807 showed higher expression in the Tianshan group and 446 showed higher expression in the Tarim group. The rumen contained 942 robust pairs (680 Tianshan-high and 262 Tarim-high), accounting for 75.18% of the study-wide total. The rectum contained 166 pairs (62 Tianshan-high and 104 Tarim-high). Together, these tissues contributed 88.43% of robust pairs. The reticulum contained 36 pairs, omasum 46, liver 27, jejunum 14, ileum 10, abomasum five, cecum four, colon two, and duodenum one (Figure 2A).

Directional enrichment returned 77 records meeting both FDR thresholds: 44 Gene Ontology (GO) terms and 33 Kyoto Encyclopedia of Genes and Genomes (KEGG) pathways. Of the 44 GO terms, 30 were associated with the rumen, 11 with the reticulum, two with the omasum, and one with the rectum. The 33 KEGG pathway records were concentrated in the rumen and reticulum (Figure 2B).

In the Tianshan-high rumen set, enriched categories converged on contractile and cytoskeletal organization, cell–matrix interaction, ion handling, and rhythmic regulation. Representative terms included muscle–cell cytoskeleton (fold enrichment 4.51; 31 overlapping genes), actin cytoskeleton (4.33; 22 genes), Z disk (5.94; 13 genes), actin binding (3.20; 23 genes), focal adhesion (5.71; 23 genes), calcium-ion transmembrane transport (5.98; 11 genes), and circadian entrainment (7.32; 17 genes) (Figure 2C). By contrast, Tarim-high genes in the reticulum were enriched for external plasma-membrane surface (23.45; 7 genes), cell-surface receptor signaling (22.39; 5 genes), immunological synapse (56.25; 5 genes), and negative regulation of T-cell receptor signaling (76.31; 4 genes) (Figure 2D). Representative terms and their membership are given in Tables S4 and S5.

### 3.3. Group-Associated Functional Units Are Predominantly Tissue-Restricted

**Figure 3:**
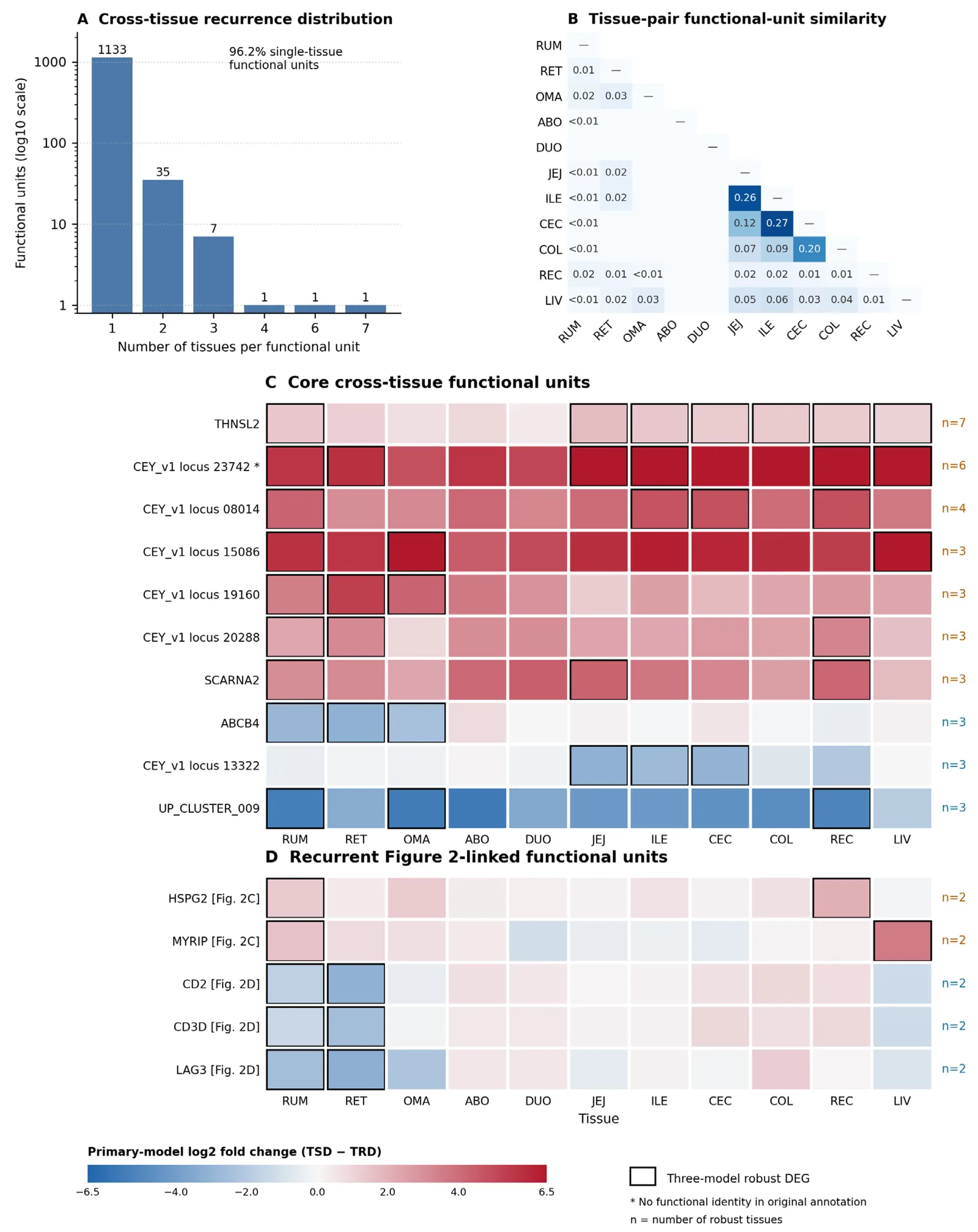
Cross-tissue recurrence and core group-associated functional units. (**A**) Distribution of the number of tissues per functional unit. (**B**) Pairwise Jaccard similarity of robust functional-unit sets; blank cells denote zero overlap. (**C**) Consistently directed units detected in at least three tissues. (**D**) Recurrent units linked to the enrichment patterns in Figure 2. Cell color represents the primary-model log_2_ fold change (TSD − TRD). Black outlines identify gene–tissue pairs meeting the robust DEG criterion in all three models; un-outlined cells show the primary-model estimate for context. The value n is the number of robust tissues. An asterisk marks the absence of functional identity in the original CEY_v1 annotation. Coordinates and exploratory BLASTp annotations are given in Table S12; the original locus identifiers remain unchanged.

Collapsing annotation-equivalent records yielded 1178 functional units. Of these, 1133 (96.18%) were robust in one tissue, 35 in two tissues, seven in three tissues, one in four tissues, one in six tissues, and one in seven tissues (Figure 3A). Therefore, only 45 units recurred in at least two tissues. Pairwise Jaccard similarities were generally low, consistent with strong anatomical localization. The largest values linked ileum with cecum (0.27), jejunum with ileum (0.26), and cecum with colon (0.20), indicating that recurrent group effects were most evident among neighboring intestinal regions (Figure 3B).

Among consistently directed units, *THNSL2* showed higher expression in the Tianshan group in seven tissues. CEY_v1 locus 23742 recurred in six tissues and locus 08014 in four; loci 15086, 19160, and 20288 and *SCARNA2* recurred in three. *SCARNA2* (ENSCHYG00000025591) is annotated as small Cajal body-specific RNA 2, a noncoding scaRNA linked to Rfam RF01268. Tarim-group recurrent signals included *ABCB4*, locus 13322, and UP_CLUSTER_009 in three tissues (Figure 3C). UP_CLUSTER_009 is an analysis-defined functional unit comprising ENSCHYG00000016367 and ENSCHYG00000016408, which share UniProt accession A0A833W3H0; it is not an official gene symbol. No additional validated functional name is assigned to the unnamed loci. *HSPG2* and *MYRIP* were higher in the Tianshan group in two tissues, whereas *CD2*, *CD3D*, and *LAG3* were higher in the Tarim group in rumen and reticulum (Figure 3D). *MSX1* had opposite directions in rumen and rectum and was excluded from the consistent-direction heatmap. Coordinates and exploratory BLASTp hits for the nine displayed loci are provided in Table S12. Both loci in UP_CLUSTER_009 matched RING finger protein 208 sequences, supporting provisional similarity-based annotation of this analysis-defined unit.

### 3.4. Whole-Genome Data Support Group-Level Structure

The whole-genome analysis included the same six Tarim and six Tianshan animals. Autosomal SNP PCA separated the recorded groups along PC1 under both DP ≥ 3 and DP ≥ 5 genotype-depth filters (Figure S2). The panels retained one SNP per 50 kb interval and contained 46,586 and 45,512 SNPs, respectively; PC1 explained 36.46% and 32.68% of variance. Group separation persisted after excluding T23001 (Figure S2). Across the full candidate SNP sets, weighted Weir–Cockerham *F*_ST_ was 0.341 for DP ≥ 3 and 0.325 for DP ≥ 5. Pairwise KING estimates were used descriptively; because a complete pedigree was unavailable, they were not used to assign specific family relationships. The full DP ≥ 3 and DP ≥ 5 candidate sets comprised 15,450,422 and 7,124,110 SNPs, respectively (Table S13). These analyses describe within-cohort genetic structure and are not a reference-panel taxonomic assignment.

## 4. Discussion

### 4.1. Conserved Tissue Programs Form the Dominant Axis of Variation

The paired design allowed group-associated expression to be evaluated against the larger transcriptional differences among organs. Tissue accounted for more than half of gene-wise variance at the median and organized both the principal-component map and correlation structure. This agrees with multi-tissue studies in humans and cattle, in which organ identity is a major determinant of steady-state transcription [7,8]. It supports estimating contrasts within each tissue rather than pooling the digestive tract.

The median population variance fraction was <0.001. Separately, population explained >5% of expression variance for 8.58% of genes, and the population-by-tissue term exceeded 5% for 9.64%. These quantities are not contradictory: the median describes the typical gene, whereas the percentages describe the subset of genes above the stated threshold. Most genes retained conserved tissue programs, while a subset showed tissue-dependent group-associated expression. The low median run-class contribution and the persistence of DEG calls across three model specifications support the robustness of the reported expression contrasts, but do not establish their genetic cause.

### 4.2. The Rumen Shows the Largest Group-Associated Expression Signal

The rumen accounted for about three quarters of robust gene–tissue pairs and contained the largest number of enriched terms. Rumen tissue responds to fermentation products, osmotic conditions, mechanical forces, epithelial turnover, and systemic metabolic cues [11,12]. Tianshan-group genes were enriched for annotations involving muscle-cell cytoskeleton, actin organization, focal adhesion, Z-disk components, and calcium-ion transport. Because the full-thickness wall was analyzed, these signals may arise from epithelial, stromal, vascular, muscular, or immune-cell differences. They should therefore be interpreted as organ-level associations rather than as evidence of a cell-specific epithelial mechanism.

Circadian entrainment was among the annotations enriched in the Tianshan rumen contrast. Feeding time, motility, and nutrient flux vary over the day, but a single collection window cannot establish a shifted or altered peripheral clock. Both groups experienced the same scheduled feeding during the common-management period, yet prior exposure and individual rhythms were not measured. Repeated, time-resolved sampling would be required to test a circadian mechanism.

The common-ration design describes group-associated expression under a shared 90-day feeding and management period. It does not show that the expression differences are caused by historical diet or local adaptation. Regional field studies report different seasonal forage contexts for Tarim and Tianshan deer [5,6], but the source history and lifetime diets of the farmed animals were not documented. The ruminal structural, adhesion, transport, and rhythmic annotations are therefore hypotheses for histological, physiological, and dietary follow-up, not established determinants of roughage utilization, health, or resilience.

### 4.3. Reticular Immune-Associated Expression Distinguishes the Tarim Group

Tarim-group genes in the reticulum were enriched for plasma-membrane localization, cell-surface receptor signaling, immunological synapse, and regulation of T-cell receptor signaling. *CD2*, *CD3D*, and *LAG3* were higher in this group in both rumen and reticulum. Full-thickness wall includes multiple cell types, so the pattern may reflect cell abundance, activation state, or local signaling. Bacterial and protozoal communities, microbial fermentation products, and dietary substrates could influence these processes [11,12,13,14,27]. However, this manuscript does not analyze matched microbiome or fermentation measurements; these mechanisms remain hypotheses. Longitudinal studies combining microbial profiles, fermentation measurements, diet records, and tissue phenotyping are needed to test them.

The strongest Tarim-group immune-associated signal was concentrated in the reticulum and selectively shared with the rumen rather than being uniformly elevated across gastrointestinal tissues. This localization is consistent with the low overlap among most tissue pairs, but it does not identify the exposure or mechanism responsible for the pattern.

### 4.4. Cross-Tissue Recurrence Reveals a Small Core Beyond Local Responses

The recurrence analysis distinguished widespread group-associated regulation from tissue-local responses. More than 96% of functional units appeared in one tissue, and the highest tissue-pair Jaccard index was 0.27. Neighboring intestinal regions showed the greatest overlap, a pattern compatible with shared developmental origin and related mucosal functions. The absolute values demonstrate that local anatomy remains a strong constraint on group differences.

The recurrent core provides candidates for mechanistic follow-up. *THNSL2* was the broadest Tianshan-group signal, spanning seven tissues, while several unannotated CEY_v1 loci showed coherent effects in three to six tissues. Their coordinates and provisional protein-similarity annotations are now supplied in Table S12. These BLASTp matches provide starting points for interpretation and do not establish the biological function of the deer loci. *ABCB4* links one recurrent signal to hepatobiliary and membrane-transport biology, while *CD2*, *CD3D*, and *LAG3* connect the two forestomach compartments through immune-associated expression. The opposite directions of *MSX1* in rumen and rectum illustrate why directional consistency matters when defining recurrence.

### 4.5. Veterinary Relevance and Study Scope

The shared feeding conditions and three-model concordance support this cohort comparison. WGS PCA and weighted *F*_ST_ describe genetic structure between the two recorded source groups, with consistent PCA separation across both depth filters and after excluding T23001. Nevertheless, the cohort includes only six adult males per group, possible within-farm relatedness, and no external taxonomic panel. KING estimates do not establish particular pedigrees. Full-thickness tissue composition, unrecorded fine landmarks outside the standardized rumen site, and four libraries with RIN below 5 may affect interpretation. A dedicated RNA-integrity sensitivity model was not fitted. Mapping both groups to a Tarim-derived reference may also contribute reference-related bias. These limitations preclude causal or population-wide claims.

The expression patterns suggest questions for veterinary and comparative physiology studies: whether ruminal cytoskeletal and adhesion annotations correspond to measurable wall structure or motility, and whether reticular immune-associated signals correspond to cell composition or local activity. The present data do not demonstrate improved health, resilience, or management utility. Testing those possibilities will require independent animals, validated taxonomic assignment, histology and immune phenotyping, cell-resolved assays, and measurements of diet, microbiome, fermentation, and clinical outcomes.

The common-environment design compares expression between two source-defined cohorts while reducing variation in the recent feeding and housing period. It cannot separate inherited differences from prenatal, developmental, early-life, or prior-habitat effects. The adult male cohort and full-thickness sampling define the scope of inference. Single-cell or spatial assays and longitudinal sampling could resolve cell composition and temporal responses in future work.

## 5. Conclusions

Across 132 matched tissue samples from 12 adult males, tissue identity was the principal determinant of gene expression. Group-associated differences were localized, with rumen and rectum contributing most robust gene–tissue pairs. Tianshan-group rumen genes were enriched for cytoskeletal, focal-adhesion, calcium-transport, and circadian-entrainment annotations; Tarim-group reticulum genes were enriched for cell-surface and immune-signaling annotations. Cross-tissue recurrence was uncommon and identified *THNSL2* and several provisional CEY_v1 loci for follow-up. These findings provide organ-level candidates for independent validation. The WGS analyses support genetic differentiation among the sampled animals under the recorded group labels. Neither analysis establishes taxonomic identity, population-wide differentiation, local adaptation, physiological resilience, or veterinary benefit.

## Figures and Tables

**Table 1:** Ingredient composition and nutrient levels of the experimental diet (air-dry basis).

Item	Proportion or Content
**Ingredie** **nts**	
Corn stover, %	19.5
Wheat straw, %	13.0
Alfalfa meal, %	22.5
Cottonseed hulls, %	15.0
Corn, %	23.5
Soybean meal, %	2.5
Cottonseed meal, %	2.5
Salt, %	0.5
Premix, %	1.0
**Calculated nutrient levels**	
Digestible energy, MJ kg^−1^	11.10
Crude protein, %	11.51
Calcium, %	0.53
Phosphorus, %	0.24

Premix supplied per kilogram of diet: vitamin A, 940 IU; vitamin E, 20 IU; S, 200 mg; Fe, 24 mg; Cu, 8 mg; Mn, 40 mg; Zn, 40 mg; I, 0.3 mg; Se, 0.2 mg; and Co, 0.1 mg. Digestible energy was calculated from ingredient values.

## Data Availability

The complete set of 156 paired-end RNA-sequencing libraries has been deposited in the NCBI Sequence Read Archive under BioProject accession PRJNA1529954 and SRA Study accession SRP736978. The archive records specify a scheduled public release date of 30 September 2028. The present organ-level analysis used 132 libraries representing 11 tissues; the additional 24 archived libraries represent rumen muscular layer and rumen papilla. Differential-expression, functional-annotation, enrichment, and figure-source tables are provided in the Supplementary Materials. The whole-genome resequencing data used for complementary population-structure analyses are not currently public. The authors plan to deposit these data with a subsequent study integrating genomic, metagenomic, and metabolomic data. Private access to the deposited RNA data and the DNA resequencing data can be provided to the editorial office and reviewers by the corresponding author. This review-access arrangement does not imply that either embargoed dataset is already publicly released.

## Supplementary Materials

The following supporting information can be downloaded at: https://www.mdpi.com/article/10.3390/vetsci13101066/s1, Supplementary_Tables.xlsx, containing Tables S1–S13; Supplementary_Figures.docx, containing Figure S1 (within-tissue RNA PCA with sequencing-run labels) and Figure S2 (complementary WGS PCA); Supplementary_Dataset_1_Expression_and_differential_expression.zip; Supplementary_Dataset_2_Functional_annotation_and_enrichment.zip; and Supplementary_Dataset_3_Figure_source_data.zip. The figure-source archive includes the PCA coordinates, Figure 2 raw counts, locus annotations, unmodified BLASTP (version 2.17.0+) XML output, search parameters and public-reference sequence provenance.
