# Supplementary material for: Multi-Tissue Transcriptomic Profiles of Tarim and Tianshan Red Deer Under Common Conditions
Source: Vet Sci. 2026 Oct 9;13(10):1066. doi: 10.3390/vetsci13101066 (PMC13709833; doi:10.3390/vetsci13101066)
Supplement: Supplementary file 1 [file vetsci-13-01066-s001.zip › Supplementary_Figures.pdf]

Supplementary Figures

Multi-Tissue Transcriptomic Profiles of Tarim and Tianshan Red Deer under Common Conditions

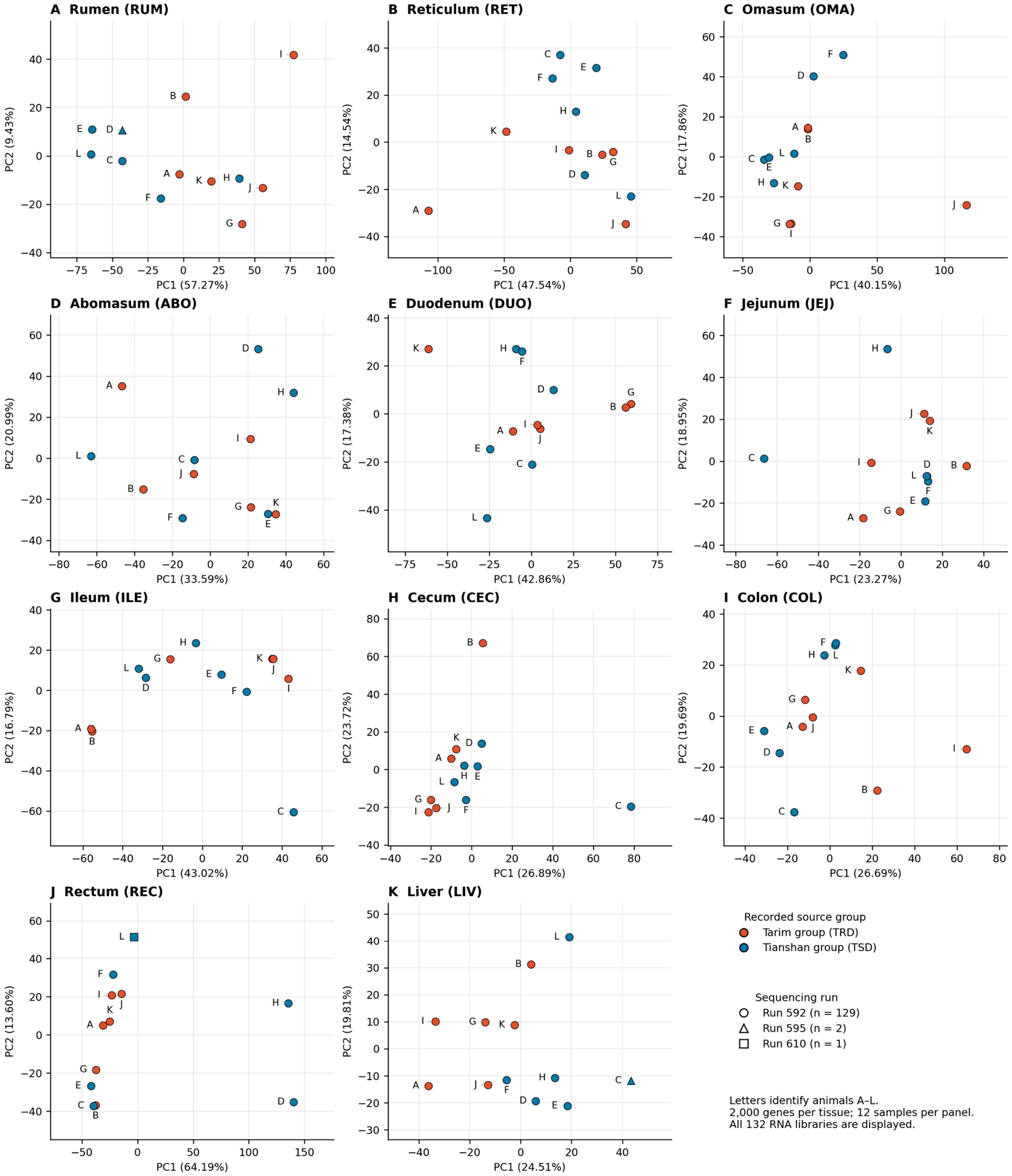

Figure S1. Within-tissue RNA principal component analysis with sequencing-run labels. Each of the 11 panels contains the 12 animals analyzed for that tissue and uses the existing 2000-gene within-tissue PCA scores. Colors identify the recorded Tarim (TRD) and Tianshan (TSD) source groups; shapes identify runs 592, 595 and 610. Letters A–L identify animals consistently across RNA and WGS panels. The 132-library dataset contains 129 samples from run 592, two from run 595 (CGZ and DLW) and one from run 610 (LZC). The main statistical models use a predominant-versus-supplemental run-class covariate. Axis percentages are specific to each tissue and are not directly comparable between panels. Coordinates and metadata are supplied in Supplementary Dataset 3.

Complementary WGS PCA

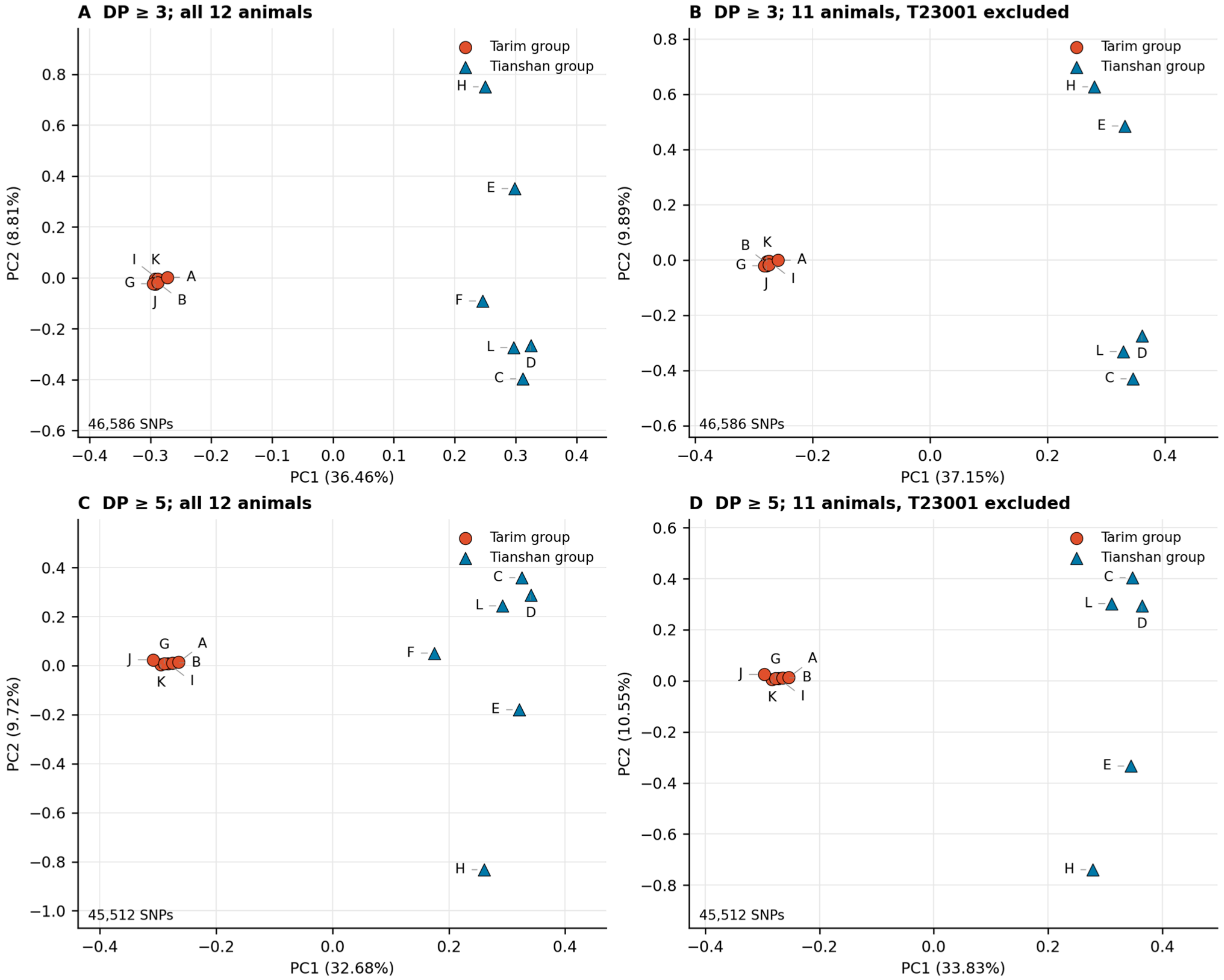

Figure S2. Complementary WGS principal component analysis across genotype-depth filters and animal subsets. (A) DP≥3, all 12 animals, 46,586 physically thinned SNPs; (B) the same DP≥3 SNP set after excluding T23001, 11 animals; (C) DP≥5, all 12 animals, 45,512 SNPs; (D) the same DP≥5 SNP set after excluding T23001. One SNP per 50-kb interval was retained and missing genotypes were mean-imputed. The complete 11- or 10-component eigenvalue spectra were used to calculate variance percentages. The 12-animal panels are primary; the 11-animal panels assess sensitivity to the elevated missingness of T23001 (animal F). Source-group colors and animal letters match Figure S1. PC signs can differ between analyses. These results describe genetic structure within this cohort; they do not establish taxonomic identity or exclude admixture. Coordinates and full variance tables are in Supplementary Dataset 3; analysis summaries are in Table S13.

Animal correspondence: A, TG092; B, TG091; C, T22005; D, T22017; E, T22039; F, T23001; G, TG061; H, T23017; I, TG044; J, TG041; K, TG031; L, T23021. RNA sample names combine the same animal letter with the tissue code. Correspondence follows the authors' animal-level records.
